# Characterization of the Gut Microbial Community of Obese Patients Following a Weight-Loss Intervention Using Whole Metagenome Shotgun Sequencing

**DOI:** 10.1371/journal.pone.0149564

**Published:** 2016-02-26

**Authors:** Sandrine Louis, Rewati-Mukund Tappu, Antje Damms-Machado, Daniel H. Huson, Stephan C. Bischoff

**Affiliations:** 1 Institute of Clinical Nutrition, University of Hohenheim, Stuttgart, Germany; 2 Algorithms in Bioinformatics, University of Tübingen, Tübingen, Germany; Western University of Health Sciences, UNITED STATES

## Abstract

**Background/Objectives:**

Cross-sectional studies suggested that obesity is promoted by the gut microbiota. However, longitudinal data on taxonomic and functional changes in the gut microbiota of obese patients are scarce. The aim of this work is to study microbiota changes in the course of weight loss therapy and the following year in obese individuals with or without co-morbidities, and to asses a possible predictive value of the gut microbiota with regard to weight loss maintenance.

**Subjects/Methods:**

Sixteen adult patients, who followed a 52-week weight-loss program comprising low calorie diet, exercise and behavioral therapy, were selected according to their weight-loss course. Over two years, anthropometric and metabolic parameters were assessed and microbiota from stool samples was functionally and taxonomically analyzed using DNA shotgun sequencing.

**Results:**

Overall the microbiota responded to the dietetic and lifestyle intervention but tended to return to the initial situation both at the taxonomical and functional level at the end of the intervention after one year, except for an increase in *Akkermansia* abundance which remained stable over two years (12.7x10^3^ counts, 95%CI: 322–25100 at month 0; 141x10^3^ counts, 95%CI: 49-233x10^3^ at month 24; p = 0.005). The *Firmicutes/Bacteroidetes* ratio was higher in obese subjects with metabolic syndrome (0.64, 95%CI: 0.34–0.95) than in the “healthy obese” (0.27, 95%CI: 0.08–0.45, p = 0.04). Participants, who succeeded in losing their weight consistently over the two years, had at baseline a microbiota enriched in *Alistipes*, *Pseudoflavonifractor* and enzymes of the oxidative phosphorylation pathway compared to patients who were less successful in weight reduction.

**Conclusions:**

Successful weight reduction in the obese is accompanied with increased *Akkermansia* numbers in feces. Metabolic co-morbidities are associated with a higher *Firmicutes/Bacteroidetes* ratio. Most interestingly, microbiota differences might allow discrimination between successful and unsuccessful weight loss prior to intervention.

## Introduction

Obesity is a growing health problem. It is associated with health disorders such as insulin resistance (IR), non-alcoholic fatty liver disease (NAFLD), or the metabolic syndrome (MetS) [1]. Apart from life style and genetics, the gut microbiota seems to play a role in obesity pathophysiology, as shown in experiments using germ-free mice displaying 40% lower body fat content than normal mice [2].

Several studies revealed different compositions of the gut microbiota between lean and obese individuals. Particularly, it was shown that a higher *Firmicutes* to *Bacteroidetes* ratio correlates with obesity [3,4]. *Firmicutes* members degrade complex polysaccharides into short chain fatty acids absorbed by colonocytes resulting in a higher energy load than *Bacteroidetes* would do [5]. However other studies failed to find such changes of the *Firmicutes* to *Bacteroidetes* ratio in obesity [6–8]. This might be due to the different techniques used for microbiota analysis, or related to differences of the patient cohorts containing patients with or without comorbidities and different obesity grades. Recently, it has been suggested that only *Bacteroidetes* numbers are regulated primarily by environmental factors such as diet, whereas *Firmicutes* numbers depend from the genetic makeup of the host [9].

A large number of studies revealed that diet, as main source of energy for the gut microbiota, has a strong impact on its composition and function (reviewed in [10]). Arumugam et al. described a limited number of well-balanced host-microbial symbioses, which are not influenced by geographical origins but by long-term dietetic habits [11]; however, gut microbiota also responds rapidly to specific changes in diet [12]. Although there have been several studies quantifying gut microbial changes after a diet intervention (reviewed in [13]), only a few involved whole genome shotgun sequencing, such as the study of Cotillard et al. [14] showing a possible predictive potential of gene richness of the microbial community for the efficacy of dietetic interventions.

To gain more insight into the interaction between microbiota, diet, and obesity we analyzed the metagenomes of 16 obese patients who underwent a weight-loss program based on a very low calorie inulin-enriched formula diet. We aimed to answer three major questions, (i) how does the microbiota change during intervention, both at taxonomic and functional level; (ii) does the gut microbiota from patients with different co-morbidities differ; and (iii) does microbiota analysis allow any prediction of successful weight-loss?

## Subjects and Methods

### Subjects and study design

For the present cohort study, we selected subjects out of a larger cohort from a multicenter clinical trial and research project "Obesity and the gastrointestinal tract" (ClinicalTrials.gov identifier: NCT01344525), approved by the ethics committee of the University Hospital of Tübingen, Germany. Written informed consent was obtained from every subject prior to participation. Exclusion criteria were chronic or current gastrointestinal disease, severe eating disorders, and treatment with anti-, pre- or probiotics within 3 months before sample collection. Selection criteria included a similar BMI and a similar age at baseline, and a subject’s affiliation to the same enterotype (*Bacteroides*-enterotype, determined through sequencing of the first sample) to minimize inter-individual variability. Among those who fulfilled these criteria, we selected individuals, who were successful regarding weight-loss maintenance after two years (relative weight loss (RWL) at T24>10%, n = 9, persistent success = PS group) or not (RWL at T24<10%, n = 7, no persistent success = NS group), as we wanted to check for potential differences in the gut microbiota between subjects with and without persistent success. A threshold of 10% weight loss and maintenance of it over one year has been proposed as definition for successful weight loss maintenance [15]. This lead to a cohort of 16 subjects (9 women) with a mean BMI of 43 ± 7 kg·m^-2^ and age of 40 ± 8 years at baseline. A predetermination of the sample size was performed for the relative weight loss (at T24) using a power of 80%, an alpha of 0.05, an expected difference between the two groups of 18% RWL and a standard deviation of 11% RWL, which lead to a group size of seven per group. The difference of 18% RWL was chosen, because we wanted it to be above the threshold of 10% RWL and higher than the observed standard deviation.

After inclusion into the study, all participants underwent a defined multidisciplinary weight-loss program over 12 months, and were further followed up for another 12 months. During the two-year-period, participants were examined at six time points, at baseline (T0), and after 3, 6, 12, 18 and 24 months (T3–T24). All participants underwent a detailed medical examination at baseline. At all time points (T0–T24), a fixed list of examinations were performed (see § clinical parameters).

### Weight-loss intervention

The multidisciplinary weight-loss program (OPTIFAST^®^ 52, Nestlé Inc.) was shown to efficiently reduce weight of obese patients and has been described in detail elsewhere [16]. Briefly, it consists of a lifestyle modification over 52 weeks based on four modules (psychology, medicine, dietetics and exercise) and includes the use of a very low calorie diet (800 kcal/day) offered as formula diet for 3 months. This formula, which is enriched with inulin as a fiber to improve bowel movements, is the only source of energy during the first three months of the program. After the three months, the formula bags are gradually substituted by normal food within a time period of eight weeks. After returning to normal food, which is a balanced diet following the recommendations of the national nutrition society, the stabilization phase starts in which patients slowly increase their energy intake to a level that allows weight maintenance. Compliance to the program for all patients was assessed by weekly visits during the whole program. The study patients followed 69% (range 29–95%) of the visits over the 12 months. Further details of the program are described elsewhere [16]. Before starting the weight reduction program, patients were asked to fill in a food diary over one week to assess dietetic habits prior to the intervention. Ten out of 16 patients provided a complete record that was analyzed using the EBISPro program [17].

### Clinical parameters

At all six time points, patients were weighed, their height and waist circumference (WC) measured. Liver sonography was performed using the LOGIQ-P6 device (GE Healthcare, Solingen, Germany) by a trained physician to assess NAFLD as described [18]. Blood was collected by venipuncture between 07.30 and 09.00 a.m. after an overnight fast. Within 15 min, serum was separated by centrifugation at 2000×g for 15 min at 4°C. Patients collected stool samples from the same passage and transferred them into collection tubes containing a DNA stabilizer (Stratec Molecular, Berlin, Germany). Blood and stool samples were stored at -80°C. Blood serum was analyzed for alanine aminotransferase (ALT), γ-glutamyl-transferase (GGT), C-reactive protein (CRP), leukocytes, fasting glucose, insulin, HbA1c, total-, LDL- and HDL-cholesterol, and triglycerides in a certified medical laboratory (Laborärzte Sindelfingen, Germany).

The Homeostasis Model Assessment-Insulin Resistance (HOMA-IR) index was calculated to estimate insulin sensitivity as described [19]. The Fatty Liver Index (FLI) is a validated marker of risk for fatty liver disease and was calculated as described [20]. We used the definition of the International Diabetes Foundation (http://www.idf.org/metabolic-syndrome) for determining the metabolic syndrome state of study participants at different time points.

The parameters were monitored in order to establish potential relationships between microbiota, body-weight, body composition, and obesity-associated-disease such as hypertension, insulin resistance,-fatty liver disease (Table 1).

**Table 1:** Clinical parameters along the study period.

Parameter	Month 0		Month 3		Month 6		Month 24	
	*mean*	*95% CI*	*mean*	*95% CI*	*mean*	*95% CI*	*mean*	*95% CI*
Body wt (kg)	129	119–140	106 ***	95,9–116	102 ***	90,8–113	116 **	104–128
BMI^1^	43,1	39,5–46,8	35,4 ***	31,8–38,9	33,8 ***	30,2–37,4	38,7 **	34,5–42,8
Waist circ. ^1^	125	116–134	105 ***	97,3–113	102 ***	93,2–110	112 **	104–120
FBS^1^ (mg/dl)	104	94,3–114	94,2 *	88,8–99,6	95,4 *	89,9–101	102	85,6–118
HbA_1C_ (%)	5,9	5,6–6,1	5,4 ***	5,3–5,6	5,5 **	5,3–5,6	5,6 *	5,3–5,8
RR dias.^1^	86,3	80,5–92,0	75,9 **	70,6–81,3	74,5 ***	69,2–79,8	79,1 *	73,5–84,6
RR sys. ^1^	127	119–136	112 **	105–120	114 **	105–122	119 **	110–128
Puls (1/min)	74,6	70,0–79,2	64,2 **	60,0–68,5	65,7 **	62,0–69,5	71,9	65,3–78,4
Total chol. ^1^	202	181–224	158 ***	141–175	178 **	159–197	189 *	166–213
HDL chol. ^1^	47,0	41,8–52,1	40,9 *	38,5–43,4	47,4	43,5–51,2	49,3	43,2–55,4
LDL chol. ^1^	129	116–144	110 *	93,4–126	114 ***	98,4–129	126	109–143
Triglycerides^1^	183	104–263	93,7 ***	72,3–115	95,3 ***	74,2–116	131 **	67,3–194
ALT^1^ (U/l)	42,2	29,6–54,7	37,8	28,0–47,6	28,9 *	14,7–43,0	33,6 *	20,4–46,8
GGT^1^ (U/l)	40,3	26,9–53,7	29,9 *	18,1–41,6	27,7 **	16,9–38,5	36,6 **	16,3–56,8
Leukocytes^1^	7,9	6,8–8,9	6,40 **	5,5–7,3	7,1 *	6,2–8,0	6,8 *	5,8–7,7
CRP^1^ (mg/dl)	9,2	6,3–12,1	10,1	2,9–17,4	5,1 ***	2,9–7,3	5,5 **	3,7–7,4
FLI^1^	94,3	87,6–101			63,7 ***	48,1–79,3	77,7 **	62,6–92,7
HOMA-IR^1^	4,8	3,3–6,2			2,1 ***	1,48–2,8	3,5	1,7–5,2
MetS^1^ (n)	9/16		6/15		4/16		7/16	
NAFLD^1^ (n)	13/16		8/16		6/16		8/16	

Statistics: Mean values and 95% confidence intervals (CI) are shown. Significant changes between baseline and month 3 (after formula diet), month 6 (after end of dietetic intervention), or month 24 (end of observation period) are indicated with *for P<0.05, **P<0.01 and ***P<0.001.

^1^Abbreviations: wt, weight; BMI, body mass index (kg/m^2^); Waist circ., waist circumference (cm); FBS, fasting blood sugar (mg/dl); RR dias. and sys., diastolic and systolic blood pressure (mmHg); Total chol., total cholesterol (mg/dl); HDL and LDL chol.; high-density lipoprotein and low-density lipoprotein cholesterol (mg/dl); Triglycerides (mg/dl); ALT and GGT, alanine aminotransferase and γ-glutamyl-transferase (U/l); Leukocytes (1/nl); CRP, c-reactive protein (ng/ml); FLI, fatty liver index; HOMA-IR, homeostatic model assessment—insulin resistance; MetS, metabolic syndrome (definition see text); NAFLD, non-alcoholic fatty liver disease (definition see text).

### Analysis of gut microbiota by shotgun sequencing

For whole metagenome analysis we used shotgun sequencing of stool DNA to assess taxonomic and functional changes at the six examination time points.

DNA extraction from the stool samples was performed using the “PSP-Spin-Stool-DNA-Plus Kit” with lyses enhancer according to the manufacturer’s instruction (Stratec Molecular, Berlin, Germany). Briefly, lysis enhancer was added to an aliquot of the samples stored in the ‘Stool DNA Stabilizer’ solution and incubated ten min. at 95°C. Next, samples were vortexed for two minutes with Zirconia beads. Cleaning of DNA was achieved first through removal of contaminants using the ‘InviAdsorb’ absorber, then by using a filter spin column. After quality check and quantification of the DNA with a Nanodrop Photometer 2000 (Thermo Scientific), DNA was sequenced on an Illumina HiSeq 2500 Sequencer by the company CeGat, Tübingen, Germany. Samples (50 ng as quantified by Qbit) were processed with the Illumina 'Nextera-DNA-Sample-Preparation Kit' according to manufacturer's protocol. Sequencing was done with 2x100 nucleotides (paired-end sequencing) on 8 lanes with 300GB raw data. On an average, the sequencing achieved 2.1 GB/sample. Samples were sequenced with a sequencing depth of 10.9 million reads per paired-end sequencing file (s = 6.3 million).

### Bioinformatic analysis of sequencing data

Raw sequences obtained from 92 metagenomic samples (16 patients, 6 time-points each, 4 time-points missing from 4 different patients) were subjected to a quality check using the FastQC software (www.bioinformatics.babraham.ac.uk/projects/fastqc/). Quality check comprising per base sequence quality, per sequence quality scores, per base sequence content, per sequence GC content, per base N content, sequence length distribution, sequence duplication levels, kmer content and over-represented sequences. All samples showed satisfactory values for each parameter tested. Next, the sequences were processed using PRINSEQ for removing low quality reads, trimming of poly-Ns and A/T tails [21]. Each sample was subjected to a BLASTX analysis using an in-house developed tool (MALT http://ab.inf.uni-tuebingen.de/software/malt/) against the NCBI-NR database with a maximum allowed e-value of 1.0. The BLASTX files were imported into MEGAN5 (http://ab.inf.uni-tuebingen.de/software/megan5/). MEGAN5 carries out binning of the reads into taxonomic and functional categories based on the BLASTX hits. The minimum bit score used for the analysis was 50 and a minimum support of 50 reads for each taxonomic category was used for the LCA algorithm. Ultimately reads get assigned to a taxonomic and functional category. On an average, about 50% of the reads in each sample was assigned to some category, 79% thereof down to the level of genera and about 61% to the level of species. The samples were normalized with respect to each other. The functional annotation of the reads was done based on the KEGG library (Kyoto Encyclopedia for Genes and Genomes, http://www.genome.jp/kegg/). Metagenomic data is available in the NCBI database under Bioproject ID PRJNA290729.

### Statistics

The normalized read counts for taxonomic and functional categories were used for all statistical tests by using the R software (http://www.r-project.org/) and its packages MASS, vegan and labdsv [22–24]. The indval function was used for detection of indicator species in ecological studies [25]. Changes over time were identified using Friedman’s test, differences between two time-points using Wilcoxon’s test for paired samples and differences between two independent groups using Mann-Whitney’s test. Correlations between microbiota components and biological parameters were analyzed using Spearman’s test. Benjamini-Hochberg’s correction (BH) was used for multiple testing. To look for possible microbial markers of weight-loss success, an Orthogonal Projections to Latent Structures-Discriminant Analysis (OPLS-DA) was performed with the SIMCA13.0 software (UmetricsAB, Umea, Sweden), using Pareto-scaling and seven-fold cross-validation [26]. A p value lower than 0.05 was considered as statistically significant.

## Results

### Effect of the weight-loss program on clinical parameters

**Fig 1:**
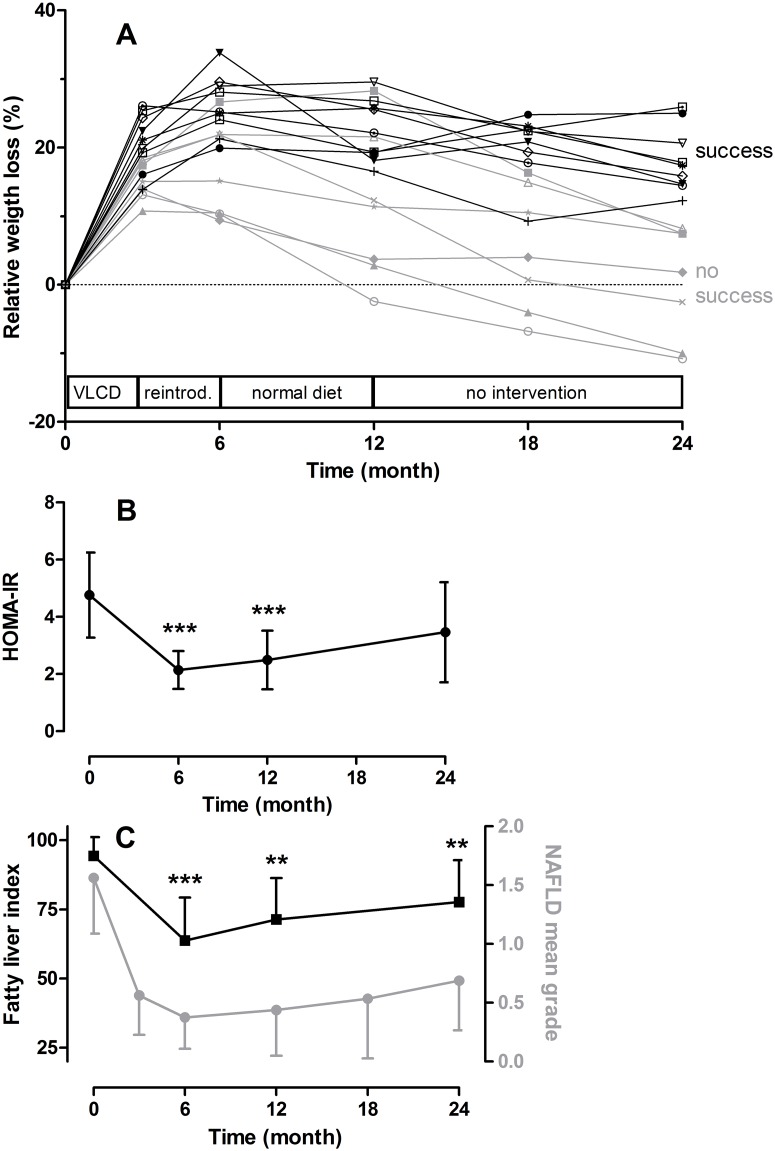
Clinical parameters of the study population. A. Relative weight loss during the observation period of two years consisting of one year intervention program with very low calorie diet (VLCD) during the first 3 months, reintroduction of normal food (reintrod.) during month 3–6, and weight maintenance therapy under normal diet during month 7–12, followed by a one-year-observation without intervention. Each line represents a patient (n = 16). Patients were grouped into those with persistent success (PS group, >10% RWL at T24, black lines and symbols) or no persistent success (NS group, <10% RWL at T24, grey lines and symbols). B. Change of insulin resistance during time. Insulin resistance was assessed using the HOMA-IR as described in Subjects and Methods. C. Change of liver steatosis assessed by sonography (circles) and fatty liver index (squares). Data in B and C are indicated as means +/- 95% confidence intervals (n = 16), **P<0.01 and ***P<0.001 (as compared to baseline, Wilcoxon’s test).

The multidisciplinary weight-loss program lead to a strong weight reduction during the first three months in all participants, and a further weight-loss during the following three months when formula diet was gradually replaced by normal food (mean relative weight loss, RWL T0/T6 = 22.0±7.3%). After the six first months, no further weight loss was observed, as expected (maintenance phase). This tendency became even more visible within the second year resulting in an average mitigation of success (Fig 1A). As expected patients fell into two categories regarding long-term weight loss; those with persistent success (RWL at T24>10% mean = 18.2 ± 4.7%, n = 9, PS group), and those with no success (RWL at T24<10% mean = 0.2 ± 8.2%, n = 7, NS group). The difference in RWL between the 2 groups was highly significant (T-test: p<0.0001). The PS and NS group showed no differences regarding age, sex, and initial BMI (not shown), however, a significant difference in the rate of visits attended during the 12-months-program was observed between the two groups. Patients with persistent success attended 80% of the visits, whereas those without persistent weight loss only attended 58% of the visits (p = 0.01). Virtually all parameters associated with obesity-related co-morbidities listed in Table 1 correlated with the BMI (not shown). Also insulin resistance (assessed by calculating the HOMA-IR) and liver steatosis (estimated by the FLI) paralleled body-weight changes over time (Fig 1B and 1C).

### Bioinformatic analysis

Over all samples, around 55 phyla, 1000 genera, 2000 species and 6000 different KEGG genes could be detected. The most abundant phyla were *Bacteroidetes* (contributing for 68% of all counts), *Firmicutes* (27%), *Proteobacteria* (1.7%), *Actinobacteria* (1.7%), and *Verrucomicrobia* (1.3%). At the genera level *Bacteroides* clearly dominated (55%), followed by *Alistipes* (8.0%), *Faecalibacterium* (6.4%), *Eubacterium* (5.4%). Among the 290 detected KEGG pathways the most abundant ones were “selenocompound metabolism” (6.0%), “tryptophan metabolism” (5.9%), “chemical carcinogenesis” (5.8%). For subsequent analyses, only taxa or functional levels with a mean relative abundance >10 counts were taken into account, which effectively were 632 genera, 1228 species and 211 pathways.

### Taxonomical analysis of the metagenomic samples

At the phylum level no significant changes could be detected over whole intervention (Friedman test); however, when selectively comparing changes between T0 and T3 some very rare phyla like *Euryarchaoteae* (p = 0.0002) and *Deinoccocus-thermus* (p = 0.0003) increased at T3.

In previous studies, the *Firmicutes*-to-*Bacteroidetes* (F/B) ratio was linked to body-weight and BMI [3,4]. Therefore, we calculated the F/B ratio for each sample and found a high variability between individuals and time-points without correlation with BMI or other clinical parameters (not shown).

To analyze the microbial community at the genera and species levels, a non-metric multi-dimensional scaling analysis was performed. Microbiota of the obese patients displayed a high inter-individual variability before start and during intervention. While the genera composition at baseline overlapped partly with the microbiota at T3, the following time-points tended to cluster between the 2 first ones, as shown for T24 (S1 Fig). Using the adonis function of the vegan package [23], we performed a permutated MANOVA (multivariate analysis of variance) to test if the variation in the microbial community could be attributed to time and/or individual. We found a significant impact only of individual (p<0.001) even if this factor could only explain a small part of the variation (sum of squares explained = 5%). No association was found between the microbial composition at the genera level and age or sex, respectively.

**Fig 2:**
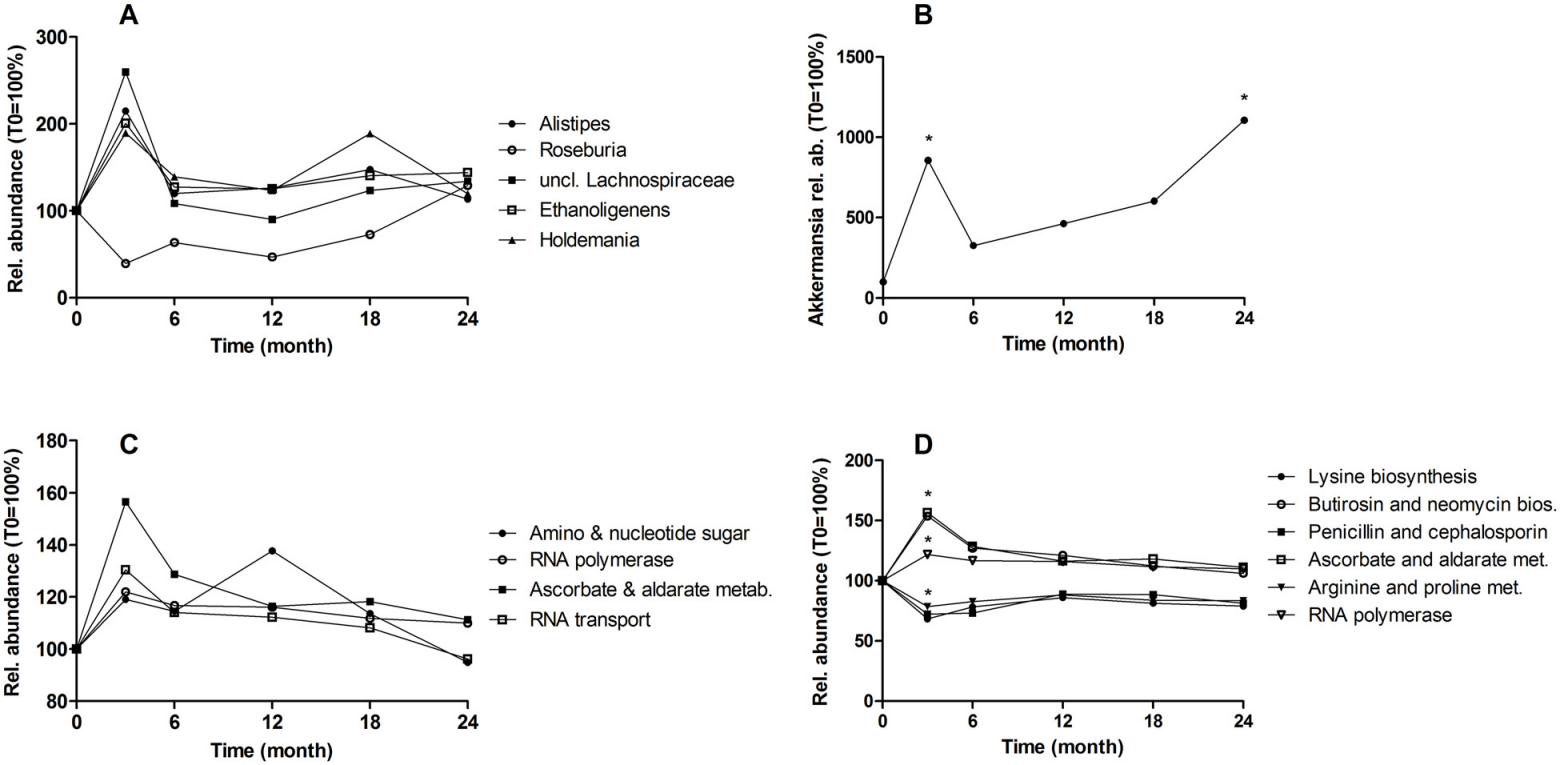
Abundance change of genera and metabolic pathways during the study. A. Relative abundance of five genera, which were the most abundant among those that changed during time. B. Akkermansia abundance. C. Relative abundance of all KEGG pathways that changed during whole study. D. Metabolic pathways that changed from baseline to T3. Abbreviations: bios, biosynthesis; met, metabolism. Statistics: Relative abundances are expressed in percent (abundance at T0 is 100%). Each dot is the mean at a given time point. Relative abundances at different time points were compared using the Friedman test (A, C: over the six time points), or the Wilcoxon test (B, D, *p < 0.05: between baseline and T3 or T24).

When analyzing relative abundances at the genera and species level, we found no significant changes over the whole intervention when correcting for multiple tests, suggesting a strong stability of the gut microbiota during intervention. However, as we were interested in genera that might be modulated by the weight-reduction program, we identified 56 genera that changed significantly according to the Friedman’s test without correction (p<0.01, S1 Table). As shown for the five most abundant ones, most changes in genera composition occurred between baseline and T3, and were transient (Fig 2A). When comparing the genera abundance between baseline and T24, only *Akkermansia* increased significantly (11 fold, from 0.26 to 2.9% of total counts, p = 0.005, Fig 2B).

**Fig 3:**
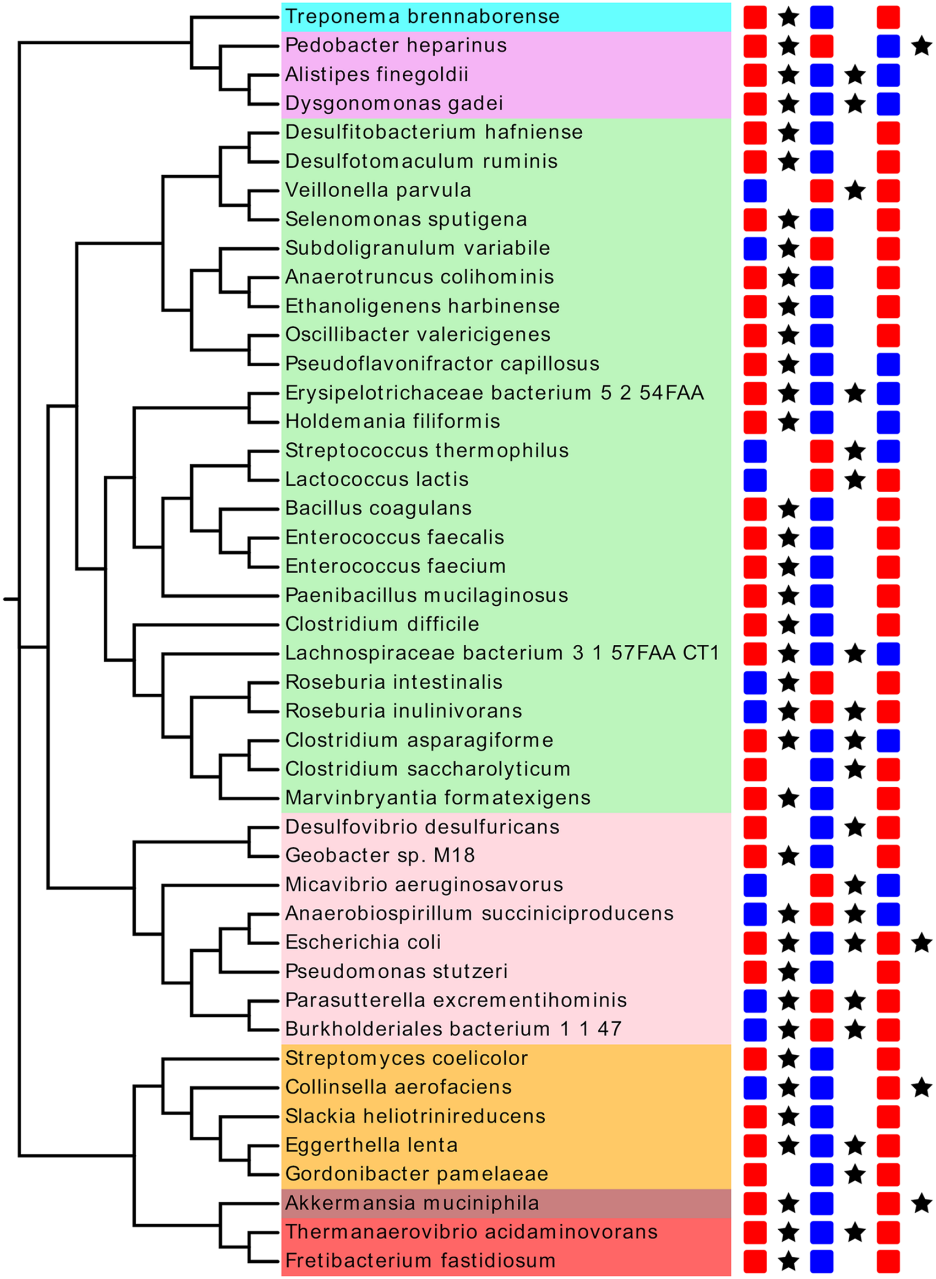
Bacterial species changes during weight loss intervention. Cladogram (based on 16S sequences) displaying the most abundant species from genera influenced by the intervention. Significant changes between T0 and T3 (left column of squares), between T3 and T6 (middle column), and between T6 and T24 (right column) are indicated by a star(p < 0.05). Blue squares indicate a decrease, red an increase in abundance. Species are colored according to the phyla they belong to (blue: Spirochaetae, pink: Bacteroidetes, green: Firmicutes, light pink: Proteobacteria, orange: Actinobacteria, brown: Verrucomicrobia, red: Synergistetes). This tree was created using the free software EvolView [27].

Also at the level of species, belonging to genera changing during intervention, we found changes in relative abundance at different time-points (Fig 3). Again, most changes were transient and comprised preferentially increases from baseline to T3. A few changes were found for species belonging to genera which did not change as a whole. For example, *Bacteroides* abundance did not change; however, that of *B*. *vulgatus*, and *B*. *dorei* decreased between T0 and T3 (both p = 0.007) whereas that of *B*. *cellulosilyticus* and *B*. *intestinalis* increased (p = 0.005 and 0.02, resp.). We also calculated the Shannon diversity index (SDI, at species level) at different time-points, because microbial gene richness was shown to have an impact on weight-loss therapy response [14]. We found a tendency of higher alpha-diversity in patients with lower BMI at T0, but not at T24 (S2 Fig). No correlation between RWL at T24 and SDI was found.

### Functional analysis of the metagenome over intervention

We could not find changes at the phyla level over time, probably because of the high variability in abundance of the five most prominent phyla between the different patients and time-points. However, functional analysis of the metagenome revealed similar clusters of orthologous groups (COG, see [28]) in all patients independent of the time-points suggesting stable functions of the microbiota despite the taxonomic variability (S3 Fig).

Furthermore, we determined changes in functional composition using the KEGG hierarchical classification at the pathway and KEGG orthologous group level. Again after correction, no significant changes were found. Without BH correction, we identified four pathways changing in abundance, the most abundant being the “Amino and nucleotide sugar metabolism” (p = 0.009, Fig 2C). When focusing on changes between baseline and T3 we found six pathways affected. The strongest effect occurred in the “Lysine biosynthesis” pathway (p = 0.0006, Fig 2D).

### Microbiota and physiological parameters in the context of the metabolic syndrome

**Fig 4:**
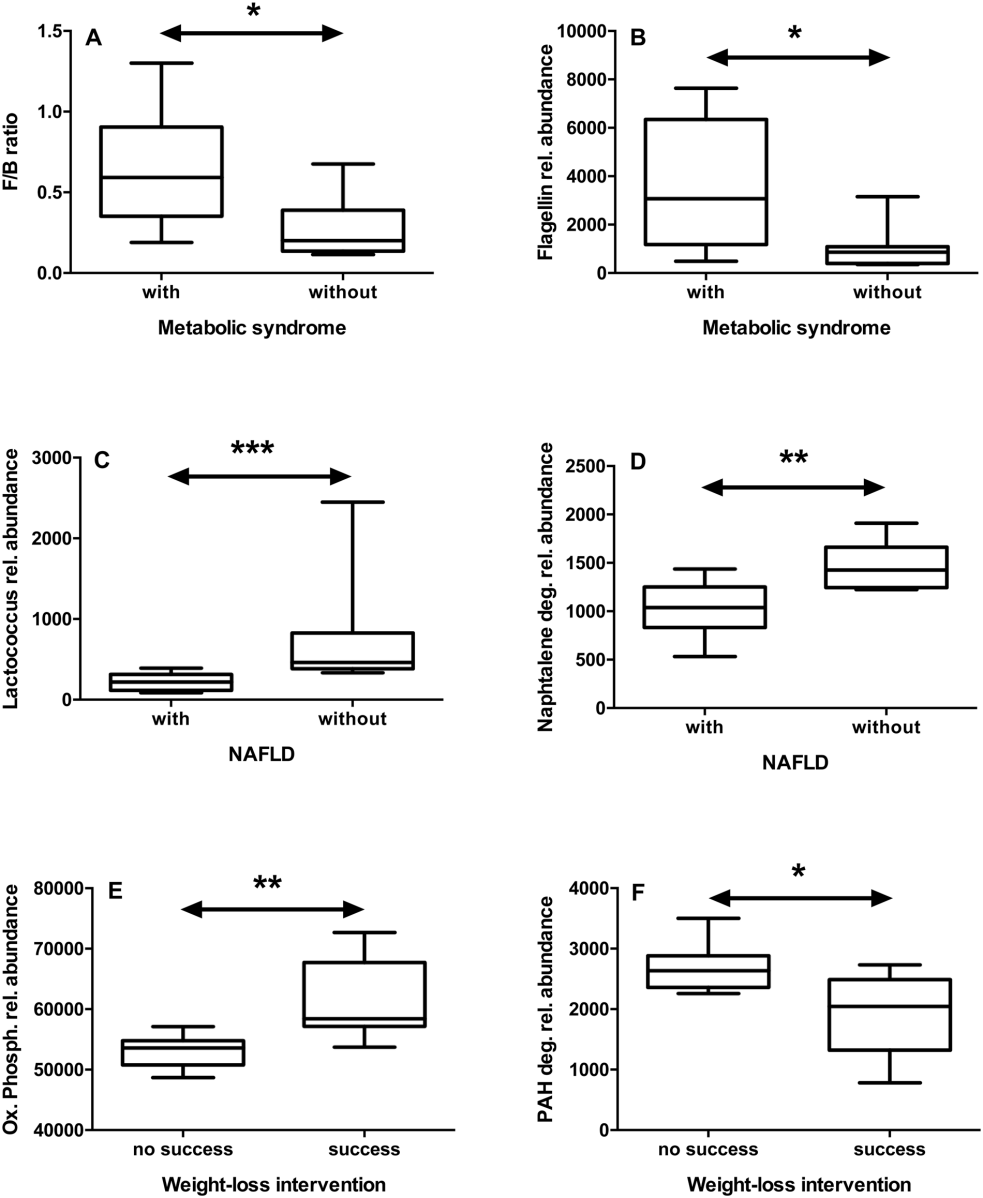
Differences in gut microbiota between patients with different co-morbidities or different outcomes. We compared patients with or without metabolic syndrome (A, B), with or without non-alcoholic fatty liver disease (NAFLD, panels C, D), and with or without persistent success in weight loss (E, F). In patients with metabolic syndrome, the *Firmicutes/Bacteroidetes* (F/B) ratio (A) and the flagellin gene (KEGG K02406) abundance (B) were increased at T0. In patients with NAFLD, the abundance of *Lactococcus* (C) and “naphthalene degradation” pathway (D) were decreased at T24. In patients with persistent success in weight loss, the abundance of the “oxidative phosphorylation” pathway was increased (E), whereas the “PAH degradation” pathway was decreased (F) at T0. Statistics: *p<0.05; **p<0.01, ***p<0.001 (Mann-Whitney’s test).

The number of patients with MetS decreased during intervention (Table 1). We compared patients with and without MetS regarding microbiota taxonomy and function. Patients with MetS had a significantly higher F/B ratio at T0 (Fig 4A), but not at T24 (not shown). In addition, we found a number of bacterial, virus and fungi taxa, that were different between patients with and without MetS, both at T0 and T24 (S2 Table).

At the functional level we could also detect differences between patients with and without MetS. At baseline, the pathways “Pentose and glucuronate interconversions”, “Two component system”, “Plant-pathogen interaction”, “Bacterial chemotaxis”, and “Flagellar assembly” (all p<0.05) including the filament Flagellin itself (Fig 4B) were more abundant in patients with MetS, in contrast to “Ascorbate and aldarate metabolism” (p = 0.04). At T24, again the pathways “Bacterial chemotaxis” (p = 0.01) and “Flagellar assembly” (p = 0.02) were more abundant in MetS participants, whereas “oxidative phosphorylation” showed the opposite pattern (p = 0.008). When correlating these differential bacterial genera and pathways with laboratory parameters related to the MetS, we found a number of highly significant correlations, both at T0 and T24, but none of them for both time-points (Table 2).

**Table 2:** Correlations between bacterial genera or pathways and parameters related to the metabolic syndrome.

Time point	*Genera* / Pathway	Parameter^1^	Rho	P value
T0	*Alistipes*	Leukocytes	-0.82	0.0002
	*Phascolarctobacterium*	ALT	0.86	< 0,0001
	Oxidative phosphorylation	Leukocytes	-0.78	0.0006
	Ascorbate and aldarate metabolism	HDL chol.	0.66	0.007
		Triglycerides	-0.66	0.007
T24	*Prevotella*	HDL	0.67	0.004
	*Subdoligranulum*	Body weight	-0.64	0.007
		HOMA-IR	-0.68	0.004
		FLI	-0.65	0.006
		CRP	-0.71	0.002
	Bacterial chemotaxis	FBS	0.64	0.008
	Flagellar assembly	FBS	0.63	0.008
		HbA_1c_	0.70	0.005

Statistics: The Spearman’s rank correlation coefficient (Rho) is shown.

^1^Abbreviations: see Table 1.

The metabolic syndrome is associated with NAFLD. We assessed NAFLD-risk by calculation of the FLI, and NAFLD-grade by sonography. Following intervention the NAFLD prevalence decreased from 81% to 40–50%. When comparing the groups of patients with and without NAFLD at T24 (n = 8 each) with respect to bacterial composition, we found several differences, e.g. a lower abundance of *Subdoligranulum* (p = 0.04) and especially *Lactococcus* (p = 0.0006, Fig 4C), while *Paraprevotella* was more abundant (p = 0.05) in the NAFLD group. We selected T24 for this analysis to exclude influences of the formula diet on the relation between NAFLD and bacterial taxa. Despite such differences, microbiota functions were largely similar. We detected only one pathway less abundant in patients with NAFLD, the “naphthalene degradation” pathway (p = 0.007, Fig 4D).

### Comparison between successful and unsuccessful patients

Comparing patient groups with persistent success (PS), *i*.*e*. with a relative weight loss above 10% at the end of the observation period, and those with no persistent success (NS) (relative weight-loss lower than 10% between T0 and T24) we found candidates for indicator genera for both groups. In the PS group, the strongest indicator values were obtained over all time-points for the two highly abundant *Akkermansia* (indval = 0.712, p = 0.02) and *Dialister* (0.702, p = 0.01). In the NS group, even stronger markers could be identified including the highly abundant *Prevotella* (0.952, p = 0.0001), *Megamonas* (0.991, p = 0.0001), *Phascolarctobacterium* (0.759, p = 0.0009) and less abundant *Barnesiella* (0.775, p = 0.04) and *Alloprevotella* (0.769, p = 0.002).

**Fig 5:**
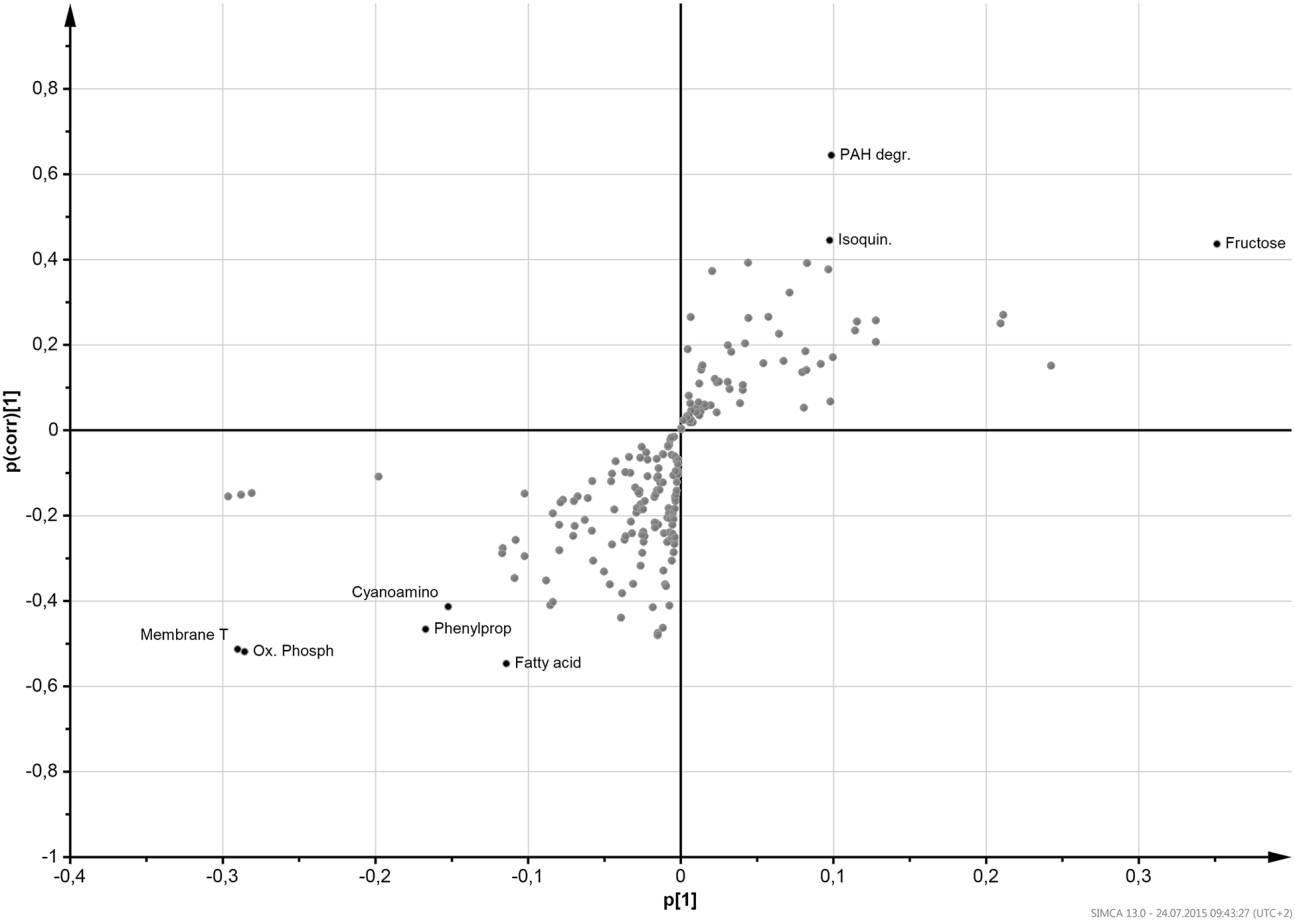
S-plot of the OPLS-DA model showing pathways associated with sustained weight loss over the whole study period. Each pathway is displayed as a dot. Dots located in the top-right corner of the figure are potential markers for non-persistent weight loss, whereas those located in the bottom-left corner are associated with sustained weight loss. On the x-axis, the loading (p|1|) is indicated, which is a measure for the influence of the variable on the model. On the y-axis, the p(corr)|1| is indicated, which is a measure of the reliability of a variable as a marker. The strongest marker (defined as |p| > 0.09 and |p(corr)| > 0.4) are labeled and displayed in black color, the other pathways are displayed without labels in grey. Abbreviations: Fructose, “fructose and mannose metabolism”; Isoquin., “isoquinoline alkaloid biosynthesis”; PAH degr.,”polycyclic aromatic hydrocarbon degradation”; Fatty acid, “fatty acid metabolism”; Phenylprop, “phenylpropanoid biosynthesis”, Cyanoamino, “cyanoamino acid metabolism”; Membrane T, “membrane transport”, Ox. Phosph, “oxidative phosphorylation” pathway. Statistics: The model is based on the following characteristics: 1+2 components, R^2^X = 0.53, R^2^Y = 0.496, Q^2^ = 0.277, pcv-ANOVA = 1.73x10^-4^. Because of low Q^2^, permutations of the data were performed before running again the OPLS-DA. This lead each time to the same model.

To search for functional markers of the microbiota that differ between PS and NS group we performed an OPLS-DA using Pareto-scaled data at the KEGG-pathway level. From this analysis over all time-points, we found two markers strongly associated with PS, “membrane transport” and “oxidative phosphorylation”, and one, “fructose and mannose metabolism” closely associated with NS (Fig 5).

To answer the question whether the microbiota at baseline could be a predictor for success, we compared bacterial taxa only at T0 in the PS and the NS group. We found no differences at the major phyla level, but numerous differences at the genera level. In the PS group, *Alistipes* (p = 0.04), *Pseudoflavonifractor* (p = 0.04), *Ethanoligenens* (p = 0.03), *Gordonibacter* (p = 0.03) and *Symbiobacterium* (p = 0.01) were more abundant. At the species level, several *Bacteroides* species such as *B*. *caccae* (p = 0.02), *B*. *sp*. 1130 (p = 0.04), *B*. *massiliensis* (p = 0.01) were less abundant in the PS group, while *Clostridium leptum* (p = 0.006), was more abundant compared to the NS group. At the functional level, we found a higher representation of the “Oxidative phosphorylation” pathway in the PS group (Fig 4E), whereas the “Cysteine and methionine metabolism” (p = 0.04) and “PAH degradation”pathways (Fig 4F) were more abundant in the NS group. Since the way of grouping of the study participants may affect the results, we performed another analysis comparing the five most successful (mean RWL at T24 = 19.5 ± 3.9%) and the five less successful (mean RWL at T24 = -2.8 ± 7.8%) patients. The participation to the program meetings was not different (74 ± 6% versus 60 ± 9%, p>0.05). Again, we found a significant enrichment in *Alistipes*, *Gordonibacter* and *Symbiobacterium* (p = 0.008 for all) and in the “Oxidative phosphorylation” pathway (p = 0.03) in the most successful patients. By trend, the “PAH degradation”pathway was more abundant in the five most unsuccessful patients than in the five most successful (p = 0.15).

Since eating behavior at baseline could influence patients ‘gut microbiota, we analyzed available food diaries. We were interested to see if patients maintaining their weight-loss successfully had an eating behavior at baseline that differs from those without sustained success. From the ten available diaries, five were from successful patients (RWL>10%). Mean (over the different days in the diary) energy, fat, protein, carbohydrate and fiber intakes were calculated as percentage of the recommended intake for each person (adapted to sex and age, as recommended by the German nutrition society). Overall, the pre-intervention-diet was too rich in protein (172% ± 24% of the recommended value) and deficient in fibers (55% ± 12%) as revealed by analysis of all ten records. We found no significant differences between the PS and the NS group regarding global energy, protein, fat, carbohydrate and fiber intake.

## Discussion

The present study shows that the intestinal microbiota is quite stable in obese individuals during non-surgical weight-reduction. In contrast to some previous literature [3,4], we could not find any consistent relation between the F/B ratio and BMI, body-weight changes or other clinical parameters. This suggests that the F/B ratio is dependent on other factors than BMI such as co-morbidities or host’s genetics [9]. Alternatively, the lack of correlation might be related to the fact that even after intervention, most study participants had a BMI above 30 kg/m^2^. The alpha diversity tended to be lower in patients with higher BMI at baseline, but not after two years, suggesting a positive effect of the intervention regarding the distal gut microbiota community.

At the genera and species levels, we found some variations related to the weight-reduction intervention. For example, we saw a reduction of the butyrate-producing *Roseburia* within the first 3 months of intervention, despite the fact that the formula was supplemented with inulin. It was shown before that low carbohydrate diet leads to a reduction of *Roseburia* and *Bifidobacteria* abundance in feces from obese subjects undergoing weight-loss [7,29,30]. In our study, *Bifidobacteria* numbers were not reduced by the formula diet, possibly because of the bifidogenic effect of inulin [31,32].

Most changes occurred transiently being maximal at month 3 suggesting a strong resilience of the gut microbiota after intervention. However, *Akkermansia* was one of the few genera that increased in abundance from baseline to the end of the program. *Akkermansia* was shown to support mice coping with high-fat diet and therefore has been proposed as an “anti-obesity bacterium” [33]. Our results confirm this, since patients had a lower mean BMI and a higher abundance of *Akkermansia* at T24 than at baseline. Moreover, *Akkermansia* is according to our data the strongest indicator for success along the intervention program, even if it does not significantly correlate with body-weight, BMI or WC at baseline and T24. Finally at T24 it is found more frequently in patients without MetS than in those with MetS further supporting the concept of *Akkermansia* reflecting a healthy intestinal microbiota [34].

While the F/B ratio did not correlate with body weight in our study, we found significant differences between participants with or without MetS. A high F/B ratio is associated with the MetS, rather than with body weight or BMI suggesting that such co-morbidities indeed affect the F/B ratio, or they are affected by it.

*Alistipes*, a *Bacteroidetes* member of the family *Rickenellaceae*, might be a bacterial genus of particular interest in the field of obesity. We found significant changes of abundance during intervention, it correlated negatively with leukocytes, and—most interestingly—it was more abundant at baseline in participants successful in losing and maintaining their weight. A higher abundance of *Alistipes* was also observed in healthy individuals compared to HIV patients treated with antiretroviral therapy or not [35,36]. On the other hand, some studies found *Alistipes* correlating with health-risk [37–39]. Possibly, only in the context of weight-loss, *Alistipes* indicates a positive constellation.

The presence or absence of NAFLD is also associated with specific patterns of the gut microbiota, both at the taxa and the functional level. For example, *Subdoligranulum* (Firmicutes, Ruminococcaceae) was underrepresented in our subjects with NAFLD, which confirm the observations of Bajaj et al. who found less *Subdoligranulum* in cirrhotic compared to healthy individuals [40]. Moreover, *Subdoligranulum* was negatively correlated at study end with different parameters related to metabolic risk such as CRP, FLI and HOMA-IR. *Subdoligranulum variabile*, the only specie of this genus, which is more abundant in patients with MetS, was shown to produce mainly butyrate and lactate from glucose [41]. Butyrate is known for its healthy potential [42,43], but as an energy source for the host, it could have rather negative effect in the context of MetS.

We found that the “flagellar assembly” pathway correlates positively with two sugar metabolism related parameters (FBS and HBA1c) and is more abundant in patients with MetS. Many flagellar products are recognized by TLR5 expressed on the intestinal mucosa. Interestingly TLR5-KO mice were shown to develop MetS [44]. Furthermore flagellar products are increased in the microbiota of these mice, which is characterized by a strong instability [45]. These mice also displayed a higher FBS level than wild-type mice. Our results tend to confirm the connection between flagellar products, MetS and sugar metabolism. Further studies are necessary to understand this connection and its mechanisms.

Most relevant, our data suggest that a particular bacterial pattern is related to subgroups of obese patients with or without persistent success. Not only *Alistipes*, but several other bacteria were associated with success. For example, *Prevotella* (Bacteroidetes) was less abundant in the successful subgroup. Not only bacterial taxa, but also metabolic pathways seem to be associated with success in weight-loss. “Oxidative phosphorylation”, the pathway leading to the production of ATP, correlated negatively with leukocytes at baseline and was more abundant in successful patients. This is surprising, since the pathway leads to the production of reactive oxygen species and is associated with inflammatory disease [46].

On the contrary, the “Polycyclic aromatic hydrocarbon (PAH) degradation” pathway yielding compounds produced during the incomplete combustion of organic matter is overrepresented in unsuccessful participants at baseline. It was shown in mice [47], and more recently in children [48], that PAH are obesogenic. A higher amount of PAH intake could therefore reduce the chances for success of the weight-loss intervention. David et al. observed a higher abundance of this pathway in the animal diet-associated microbiota [12]. Possibly, the high representation of this pathway in “unsuccessful” individuals is a result of high charred meat consumption before intervention, but other environmental sources of PAH cannot be excluded. From the available food diaries we can confirm that all participants consumed high amounts of meat, but we cannot distinguish between charred meat and other meat.

The differences in microbiota composition before intervention between successful and non-successful participants with regard to weight reduction and maintenance warrant further studies to confirm the predictive value of the microbial markers identified in the present study. Such predictors for success could help adapting weight-loss strategies individually to the obese patient and thus make obesity treatment more successful in future.

## Supporting Information

S1 Fig Non-metric Multidimensional Scaling on taxonomic composition of all samples. NMDS was performed using Bray-Curtis distance to represent all samples based on their taxonomic composition (genera level). Samples are red at T0, black at T3, and grey at T6, T12, T18 and T24. Ellipses represent mean ± SD for red: T0, black: T3 and green: T24.
(TIF)

S2 Fig Shannon diversity Index and body mass index (kg/m^2^). SDI (at the species level, for each sample at one time point) tends to negatively (Spearman’s rho = -0.37; p = 0.17) correlates with BMI at T0 (A) but not at T24 (B, p = 0.47).
(TIF)

S3 Fig Taxonomic and functional composition of the distal gut microbiota. A, B, C: Phyla; D, E, F: COG functional categories. A, D: at T0; B, E: at T3; C, F: at T24. Relative abundance is given as percentage of the whole community. J: Translation, ribosomal structure and biogenesis, K: Transcription, L: Replication, recombination and repair, V: Defense mechanisms, T: Signal transduction mechanisms, M: Cell wall/membrane/envelope biogenesis, U: Intracellular trafficking, secretion, and vesicular transport, O: Posttranslational modification, protein turnover, chaperones, C: Energy production and conversion, G: Carbohydrate transport and metabolism, E: Amino acid transport and metabolism, F: Nucleotide transport and metabolism, H: Coenzyme transport and metabolism, I: Lipid transport and metabolism, P: Inorganic ion transport and metabolism, R: General function prediction only.
(TIF)

S1 Table List of all genera influenced by the intervention. (DOCX)

S2 Table Differences in distal gut microbiota composition in obese patients with metabolic syndrome compared to patients without metabolic syndrome*. (DOCX)
