# Supplementary figures and images for: Characterization of the Gut Microbial Community of Obese Patients Following a Weight-Loss Intervention Using Whole Metagenome Shotgun Sequencing
Source: PLoS One. 2016 Feb 26;11(2):e0149564. doi: 10.1371/journal.pone.0149564 (PMC4769288; doi:10.1371/journal.pone.0149564)

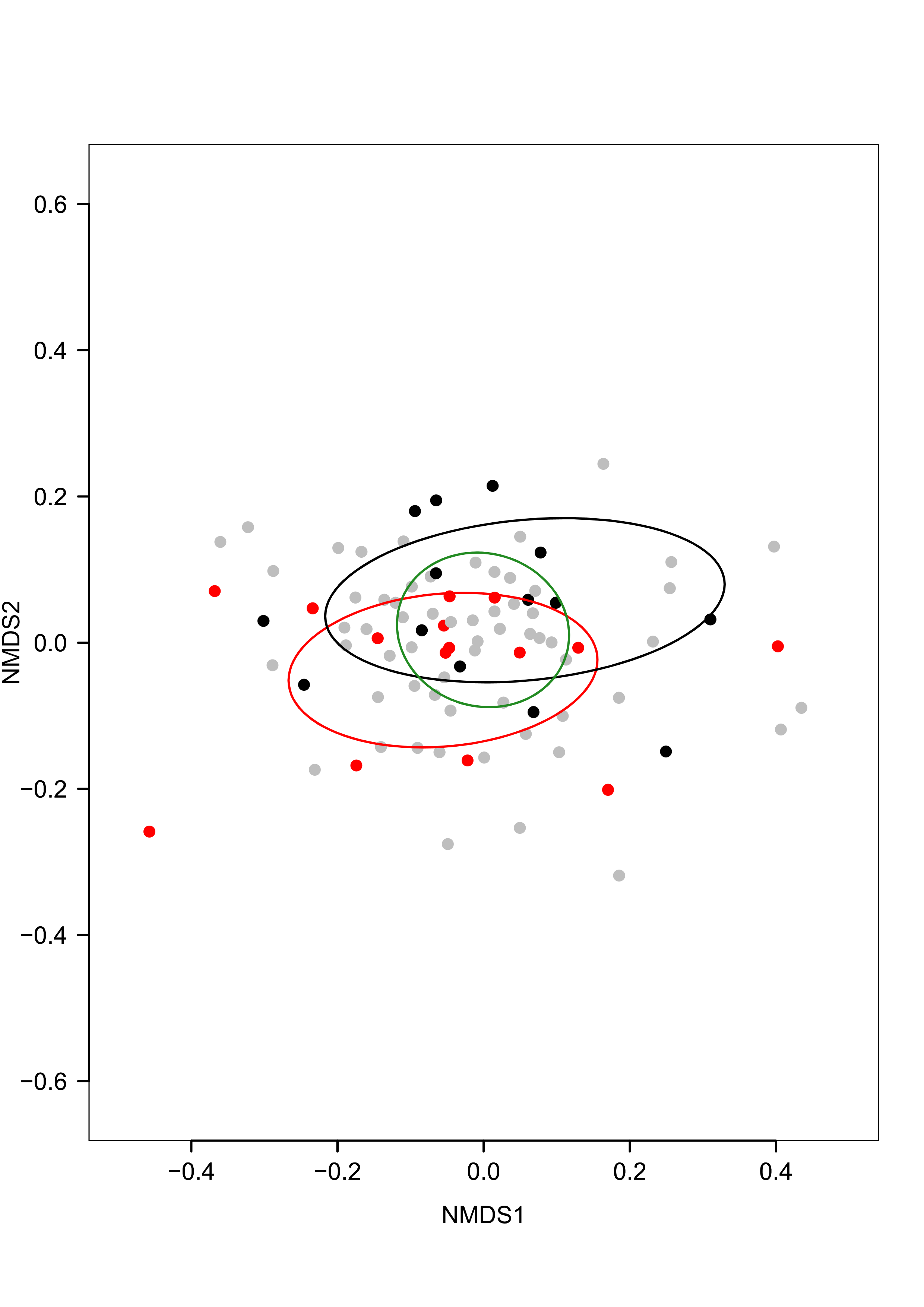

Supplement: S1 Fig — NMDS was performed using Bray-Curtis distance to represent all samples based on their taxonomic composition (genera level). Samples are red at T0, black at T3, and grey at T6, T12, T18 and T24. Ellipses represent mean ± SD for red: T0, black: T3 and green: T24. (TIF) [file pone.0149564.s001.tif]

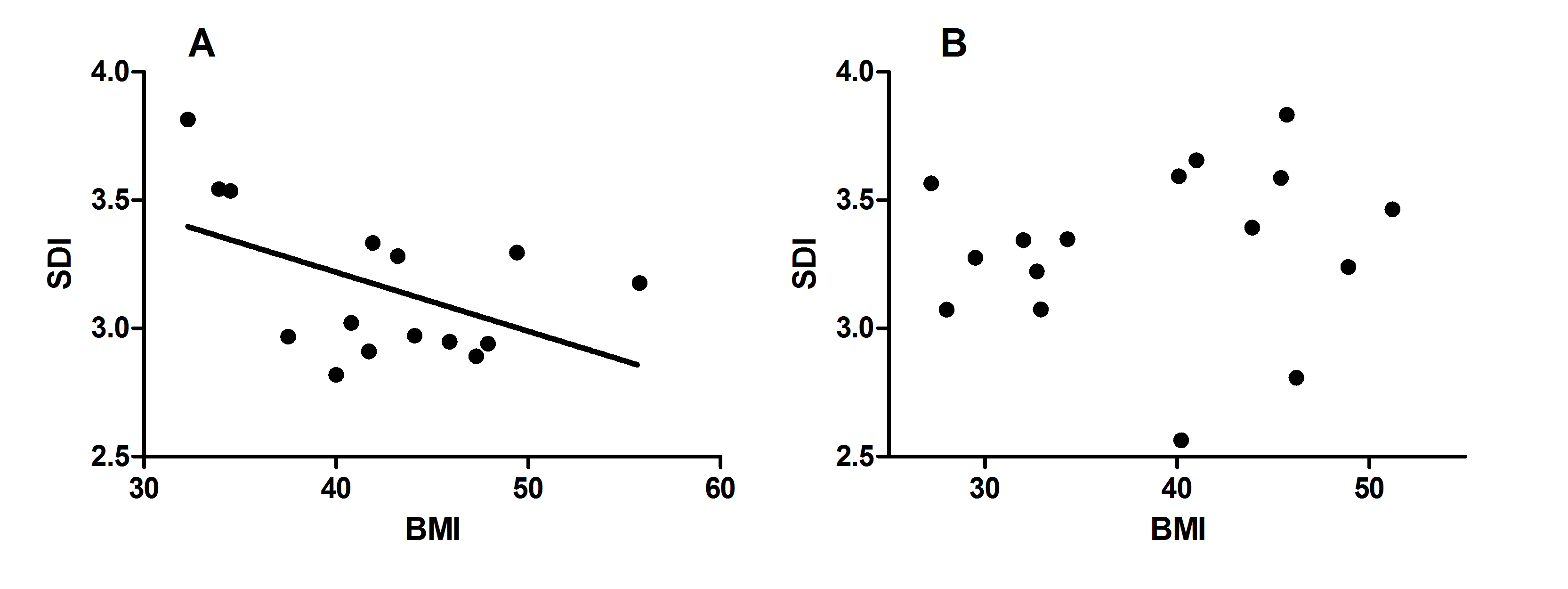

Supplement: S2 Fig — SDI (at the species level, for each sample at one time point) tends to negatively (Spearman’s rho = -0.37; p = 0.17) correlates with BMI at T0 (A) but not at T24 (B, p = 0.47). (TIF) [file pone.0149564.s002.tif]

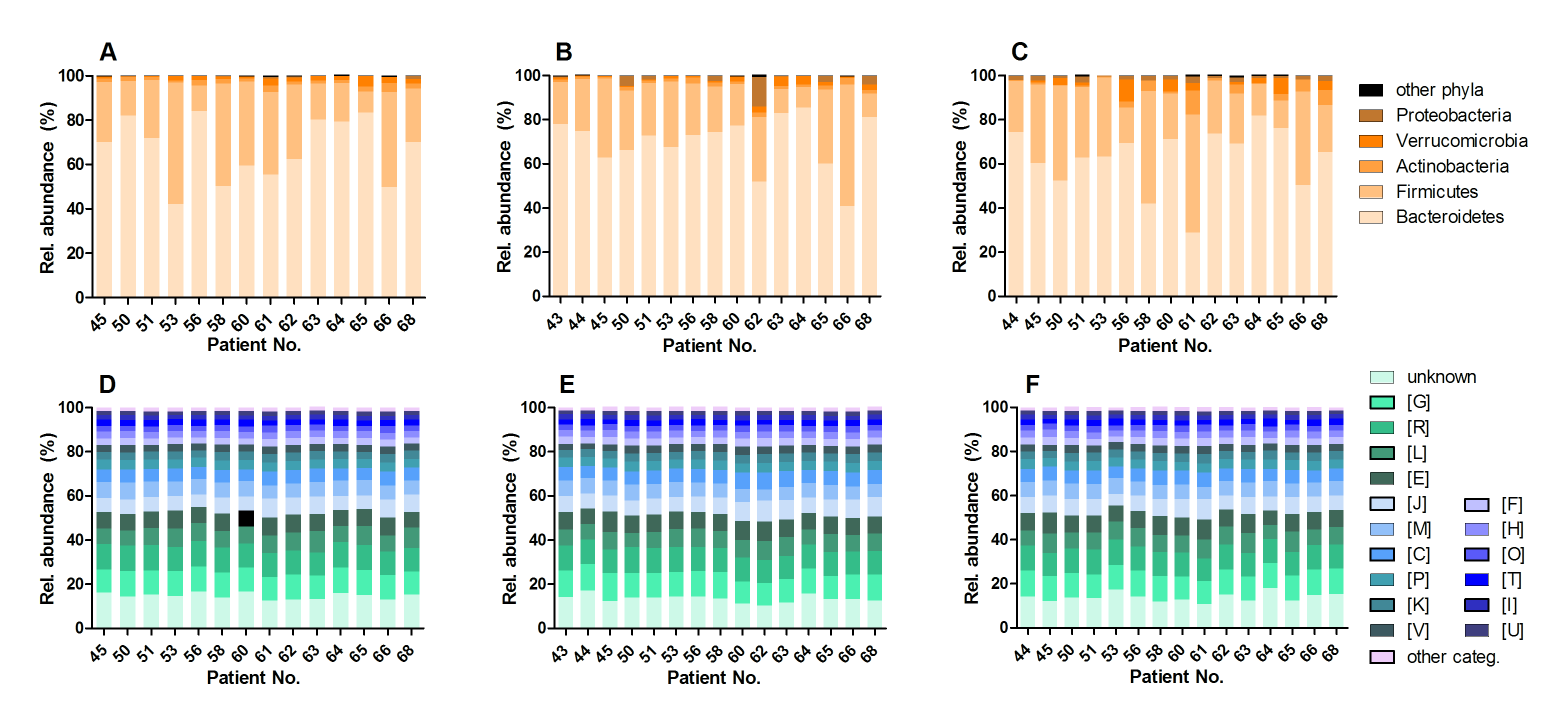

Supplement: S3 Fig — A, B, C: Phyla; D, E, F: COG functional categories. A, D: at T0; B, E: at T3; C, F: at T24. Relative abundance is given as percentage of the whole community. J: Translation, ribosomal structure and biogenesis, K: Transcription, L: Replication, recombination and repair, V: Defense mechanisms, T: Signal transduction mechanisms, M: Cell wall/membrane/envelope biogenesis, U: Intracellular trafficking, secretion, and vesicular transport, O: Posttranslational modification, protein turnover, chaperones, C: Energy production and conversion, G: Carbohydrate transport and metabolism, E: Amino acid transport and metabolism, F: Nucleotide transport and metabolism, H: Coenzyme transport and metabolism, I: Lipid transport and metabolism, P: Inorganic ion transport and metabolism, R: General function prediction only. (TIF) [file pone.0149564.s003.tif]
