# Supplementary material for: Characterization of the Gut Microbial Community of Obese Patients Following a Weight-Loss Intervention Using Whole Metagenome Shotgun Sequencing
Source: PLoS One. 2016 Feb 26;11(2):e0149564. doi: 10.1371/journal.pone.0149564 (PMC4769288; doi:10.1371/journal.pone.0149564)
Supplement: S1 Table — (DOCX) [file pone.0149564.s004.docx]

**SI-Table 1: List of all genera influenced by the intervention**

| **Genera** | **uncorrected p-value** | **Change profile** | **Mean relative abundance** |
| --- | --- | --- | --- |
| Alistipes | 0,0056 | ↑↓ | 389755 |
| Roseburia | 0,0090 | ↓↑ | 103097 |
| Unclassified Lachnospiraceae | 0,0013 | ↑↓ | 12090 |
| Ethanoligenens | 0,0067 | ↑↓ | 2712 |
| Holdemania | 0,0062 | ↑↓ | 2095 |
| Symbiobacterium | 0,0044 | ↑↓ | 678 |
| Dysgonomonas | 0,0042 | ↑↓ | 584 |
| Heliobacterium | 0,0021 | ↑↓ | 552 |
| Chitinophaga | 0,0087 | ↑↓ | 526 |
| Deinococcus | 0,0025 | ↑↓ | 508 |
| Thermaerobacter | 0,0004 | ↑↓ | 451 |
| Thermobacillus | 0,0063 | ↑↓ | 430 |
| Anaeromyxobacter | 0,0074 | ↑↓ | 430 |
| Myxococcus | 0,0020 | ↑↓ | 353 |
| Thermus | 0,0024 | ↑↓ | 321 |
| Alicyclobacillus | 0,0100 | ↑↓ | 282 |
| Coprobacillus | 0,0046 | ↑↓↑↓ | 262 |
| Rhodothermus | 0,0031 | ↑↓ | 234 |
| Robiginitalea | 0,0020 | ↑↓ | 227 |
| Moorella | 0,0060 | ↑↓ | 208 |
| Pelotomaculum | 0,0009 | ↑↓ | 205 |
| Actinoplanes | 0,0010 | ↑↓ | 201 |
| Desulfarculus | 0,0012 | ↑↓ | 194 |
| Thermanaerovibrio | 0,0033 | ↑↓ | 167 |
| Corallococcus | 0,0031 | ↑↓ | 160 |
| Candidatus,Desulforudis | 0,0096 | ↑↓ | 155 |
| Thermacetogenium | 0,0015 | ↑↓ | 153 |
| Nocardiopsis | 0,0031 | ↑↓ | 152 |
| Opitutus | 0,0040 | ↑↓ | 143 |
| Haliangium | 0,0067 | ↑↓ | 141 |
| Desulfococcus | 0,0037 | ↑↓ | 139 |
| Acetonema | 0,0080 | ↑↓ | 128 |
| Streptosporangium | 0,0089 | ↑↓ | 125 |
| Nocardia | 0,0100 | ↑↓ | 123 |
| Stigmatella | 0,0017 | ↑↓ | 117 |
| Oceanithermus | 0,0011 | ↑↓ | 115 |
| Thermomonospora | 0,0030 | ↑↓ | 111 |
| Rhodococcus | 0,0088 | ↑↓ | 110 |
| Cyanobium | 0,0044 | ↑↓ | 107 |
| Rubrobacter | 0,0011 | ↑↓ | 106 |
| Desulfurivibrio | 0,0090 | ↑↓ | 106 |
| Xanthobacter | 0,0018 | ↑↓ | 101 |
| Sphaerobacter | 0,0031 | ↑↓ | 97 |
| Ammonifex | 0,0021 | ↑↓ | 94 |
| Unclassified Siphoviridae | 0,0093 | ↓↑ | 94 |
| Desulfurispirillum | 0,0007 | ↑↓ | 91 |
| Kitasatospora | 0,0023 | ↑↓ | 90 |
| Alkalilimnicola | 0,0057 | ↑↓ | 90 |
| Kyrpidia | 0,0089 | ↑↓ | 89 |
| Catenulispora | 0,0023 | ↑↓ | 87 |
| Salinibacter | 0,0062 | ↑↓ | 75 |
| Saccharothrix | 0,0089 | ↑↓ | 59 |
| Mucilaginibacter | 0,0026 | ↑↓ | 47 |
| Truepera | 0,0045 | ↑↓ | 44 |
| Haloplasma | 0,0073 | ↑↓ | 25 |
| Dethiobacter | 0,0075 | ↑↓ | 24 |

Profile: most genera increased in abundance during the first months then return between T3 and T6 to baseline levels (↑↓), few displayed opposite variations (↓↑) or two successive increases (↑↓↑↓). Statistics: Friedman’s test, significance threshold: p-value<0.01
