# Supplementary material for: Characterization of the Gut Microbial Community of Obese Patients Following a Weight-Loss Intervention Using Whole Metagenome Shotgun Sequencing
Source: PLoS One. 2016 Feb 26;11(2):e0149564. doi: 10.1371/journal.pone.0149564 (PMC4769288; doi:10.1371/journal.pone.0149564)
Supplement: S2 Table — (DOCX) [file pone.0149564.s005.docx]

**SI-Table 2. Differences in distal gut microbiota composition in obese patients with metabolic syndrome compared to patients without metabolic syndrome***

A. At study baseline

| **Phyla (p-value)** | **Family** | **Genera** | ***Species*** |
| --- | --- | --- | --- |
| **Bacteroidetes** | Prevotellaceae | Prevotella | *P. dentalis* (0.02) |
| **(0.04)** | (0.03) | (0.04) | *P. denticola* (0.01) |
|  |  |  | *P. bivia* (0.01) |
|  |  |  | *P. disiens* (0.03) |
|  |  | Alloprevotella (0.002) | *A. tannerae* (0.002) |
|  | Porphyromonadaceae | Tannerella (0.03) | *T. forsythia* (0.03) |
|  |  | Porphyromonas | *P. gingivalis* (0.04) |
|  |  | (0.006) | *P. asaccharolytica* (0.002) |
|  | Chitinophagaceae | Chitinophaga (0.04) | *C. pinensis* (0.04) |
|  | Bacteroidaceae | Bacteroides | *B. pectinophilus* (0.01) |
|  |  |  | *B. finegoldii* (0.02) |
|  |  |  | *B. sp1130* (0.004) |
|  |  |  |  |
| **Firmicutes** | Ruminococcaceae | Subdoligranulum (0.03) | *S. variabile* (0.03) |
| **(0.02890)** | (0.01398) | Ruminococcus | *R. bromii* (0.04) |
|  |  |  | *R. albus* (0.04) |
|  |  |  | *R. lactaris* (0.04) |
|  |  |  | *R. flavefaciens* (0.04) |
|  | Clostridiaceae | Clostridium (0.03) | *C. saccharolyticum* (0.01) |
|  | (0.02890) |  | *C. phytofermentans* (0.03) |
|  |  |  | *C. scindens* (0.03) |
|  |  | Faecalibacterium (0.02) | *F. prausnitzii* (0.02) |
|  | Lachnospiraceae | Butyrivibrio (0.02) | *B. proteoclasticus* (0.04) |
|  |  |  | *B. crossotus* (0.006) |
|  |  | Cellulosilyticum (0.03) | *C. lentocellum* (0.02) |
|  |  | Anaerostipes (0.02) |  |
|  |  |  | *Lachnospiraceae oral tax. 107* (0.04) |
|  | Eubacteriaceae | Eubacterium | *E. ventriosum* (0.03) |
|  |  |  |  |
| **Actinobacteria** | Coriobacteriaceae | Atopobium (0.05) |  |
|  |  |  |  |
| **Spirochaetae** | Spirochaetaceae | Trepanoma | *T. succinifaciens* (0.02) |
| **Viruses** (0.04) | *Polyomaviridae (0.009)* | Polyomavirus (0.009) |  |
|  | *Retroviridae (0.01)* | Lentivirus (0.007) | *Hum. endog. retrovirus K (0.02)* |
|  |  | Deltaretrovirus (0.02) | *Hum. endog. retrovirus H (0.01)* |
|  | *Herpesviridae (0.004)* |  | *Cercopithecine herpesvirus 5 (0.004)* |
|  | *Papillomaviridae (0.006)* | Alphapapillomavirus (0.006) | *Alphapapillomavirus (0.006)* |
| **Basidiomycota (**0.008) | Malasseziaceae (0.007) | Malassezia (0.007) |  |

B. At the end of the study (2 years after baseline).

| **Phyla (p-value)** | **Family** | **Genera** | ***Species*** |
| --- | --- | --- | --- |
| **Bacteroidetes** | Rikenellaceae | Alistipes | *A. putredinis* (0.03) |
|  | Bacteroidaceae | Bacteroides | *B. massiliensis* (0.03) |
| **Firmicutes** | Bacillaceae | Lysinibacillus (0.04) |  |
|  | Ruminococcaceae | Ruminococcus | *R. torques* (0.03) |
|  |  |  | *R. obeum* (0.02) |
|  |  |  | *R. flavefaciens* (0.01) |
|  | Acidaminococcaceae | Acidaminococcus (0.02) | *A. intestine* (0.02) |
|  | Veillonellaceae | Veillonella (0.008) | *V. parvula* (0.002) |
|  | Clostridiaceae | Clostridium | *C. spSY8519* (0.01) |
|  |  |  | *C. phytofermentans* |
|  |  |  | *C. perfringens* (0.04) |
|  |  |  | *C. saccharo-perbutylacetonicum* (0.03) |
|  | Lachnospiraceae | Roseburia (0.02) | *R. hominis* (0.008) |
|  | (0.023) | Butyrivibrio | *B. fibrisolvens* (0.02) |
|  |  | Coprococcus | *C. catus* (0.008) |
|  | Eubacteriaceae | Eubacterium | *E. rectale* (0.04) |
|  | Streptococcaceae | Streptococcus | *S. parasanguinis* (0.04) |
|  | uncl. Clostridiales (0.003) | Blautia (0.02) |  |
|  |  | uncl. Clostridiales miscellaneous (0.0003) | *butyrate pr. bacterium SSC/2* (0.03) |
|  |  |  | *butyrate pr. bacterium SS3/4* (0.001) |
| **Verrucomicrobia (-0.003)** | Verrucomicrobiaceae (0.003) | Akkermansia (0.003) | *A. muciniphila* (0.003) |
| **Gamma-Proteobacteria** | Pasteurellaceae (0.011) | Haemophilus (0.04) |  |
|  | Moraxellaceae | Acinetobacter (0.05) |  |
| **Actinobacteria** | Actynomycetaceae | Arcanobacterium (0.04) |  |

*In green: taxa more abundant in patients without MetS, in red taxa more abundant in patients with MetS. Statistics: Mann Withney’s test. Tax., taxon; Hum., human; endog., endogenous; uncl., unclassified; pr., producing.
